# Modulation of Inflammatory Mediators by Polymeric Nanoparticles Loaded with Anti-Inflammatory Drugs

**DOI:** 10.3390/pharmaceutics13020290

**Published:** 2021-02-23

**Authors:** Gloria María Pontes-Quero, Lorena Benito-Garzón, Juan Pérez Cano, María Rosa Aguilar, Blanca Vázquez-Lasa

**Affiliations:** 1 Group of Biomaterials, Department of Polymeric Nanomaterials and Biomaterials, Institute of Polymer Science and Technology, ICTP-CSIC, Juan de la Cierva 3, 28006 Madrid, Spain; gpontes@ictp.csic.es (G.M.P.-Q.); bvazquez@ictp.csic.es (B.V.-L.); 2 Alodia Farmacéutica SL, Santiago Grisolía 2 D130/L145, 28760 Madrid, Spain; jperez@alodiafarmaceutica.com; 3 Networking Biomedical Research Centre in Bioengineering, Biomaterials and Nanomedicine, CIBER-BBN, 28029 Madrid, Spain; 4 Faculty of Medicine, University of Salamanca, 37007 Salamanca, Spain

**Keywords:** celecoxib, tenoxicam, dexamethasone, osteoarthritis, inflammatory mediators, nanoparticles

## Abstract

The first-line treatment of osteoarthritis is based on anti-inflammatory drugs, the most currently used being nonsteroidal anti-inflammatory drugs, selective cyclooxygenase 2 (COX-2) inhibitors and corticoids. Most of them present cytotoxicity and low bioavailability in physiological conditions, making necessary the administration of high drug concentrations causing several side effects. The goal of this work was to encapsulate three hydrophobic anti-inflammatory drugs of different natures (celecoxib, tenoxicam and dexamethasone) into core-shell terpolymer nanoparticles with potential applications in osteoarthritis. Nanoparticles presented hydrodynamic diameters between 110 and 130 nm and almost neutral surface charges (between −1 and −5 mV). Encapsulation efficiencies were highly dependent on the loaded drug and its water solubility, having higher values for celecoxib (39–72%) followed by tenoxicam (20–24%) and dexamethasone (14–26%). Nanoencapsulation reduced celecoxib and dexamethasone cytotoxicity in human articular chondrocytes and murine RAW264.7 macrophages. Moreover, the three loaded systems did not show cytotoxic effects in a wide range of concentrations. Celecoxib and dexamethasone-loaded nanoparticles reduced the release of different inflammatory mediators (NO, TNF-α, IL-1β, IL-6, PGE_2_ and IL-10) by lipopolysaccharide (LPS)-stimulated RAW264.7. Tenoxicam-loaded nanoparticles reduced NO and PGE_2_ production, although an overexpression of IL-1β, IL-6 and IL-10 was observed. Finally, all nanoparticles proved to be biocompatible in a subcutaneous injection model in rats. These findings suggest that these loaded nanoparticles could be suitable candidates for the treatment of inflammatory processes associated with osteoarthritis due to their demonstrated in vitro activity as regulators of inflammatory mediator production.

## 1. Introduction

Osteoarthritis (OA) is the most prevalent joint condition causing pain and physical disability in affected patients. It is characterized by the inflammation of the synovial joint, the progressive degradation of cartilage and alterations in the subchondral bone [1]. Cartilage degradation is the hallmark of OA, and the lack of intrinsic cartilage healing capacity makes the progression of OA an irreversible process. The inflammatory response is also crucial for the initiation and development of OA [2]. In response to a sustained inflammatory activation, chondrocytes and immune cells, such as macrophages, trigger the release of proinflammatory mediators like interleukin-1β (IL-1β), IL-6, IL-8, tumor necrosis factor-α (TNF-α), reactive oxygen species (ROS) and nitric oxide (NO) [3,4,5,6]. These inflammatory molecules increase the production of proteolytic enzymes (matrix metalloproteinases (MMP), A disintegrin and metalloproteinase with thrombospondin motifs, ADAMTS), leading to the degradation of collagen and aggrecan cartilage components [7]. In turn, degraded cartilage fragments further stimulate inflammation, generating a damaging cycle that promotes OA progression. These inflammatory mediators are, therefore, key targets for therapeutic strategies in the treatment of OA [8].

The initial treatment of OA patients with mild to moderate symptoms is based on oral, topical or intra-articularly administered anti-inflammatory drugs, including nonsteroidal anti-inflammatory drugs (NSAIDs), selective cyclooxygenase 2 (COX-2) inhibitors (coxibs) and glucocorticoids [9]. NSAIDs, which are the most prescribed drugs worldwide, owe their potent analgesic and anti-inflammatory effects to the unspecific inhibition of COX enzymes, which take part in the biosynthesis of both physiological and inflammatory prostaglandins [10]. On the other hand, coxibs specifically inhibit the COX-2 enzyme, which synthesizes prostaglandins related to inflammation, pain and fever. Beside this COX-dependent anti-inflammatory pathway, NSAIDs may exert their effect through their interaction with transcription factors, such as nuclear factor kappa B (NF-κB) or the activator protein 1 (AP-1), both regulators of the expression of various proinflammatory genes [11]. In the case of glucocorticoids, they predominantly exert their anti-inflammatory effect by switching off multiple inflammatory genes encoding inflammatory mediators such as cytokines, chemokines, adhesion molecules, inflammatory enzymes, receptors and proteins, that have been activated during a chronic inflammatory process [12].

Celecoxib (CLX, a selective COX-2 inhibitor), tenoxicam (TNX, a traditional NSAID) and dexamethasone (DEX, a glucocorticoid) are some of the most frequently used anti-inflammatory drugs for the treatment of OA in the clinic. CLX was the first COX-2 inhibitor approved by the FDA (U.S Food and Drug Administration) for the treatment of OA and rheumatoid arthritis, due to its good selectivity for the COX-2 enzyme and for its gastrointestinal tolerability [13]. TNX is used for postoperative analgesia because of its prolonged half-life and potent immediate effect [14]. Finally, DEX is a long-lasting glucocorticoid widely used for the treatment of multiple inflammatory-related diseases such as OA, since it is one of the most potent glucocorticoids available [15]. Although these drugs exert their anti-inflammatory activity at different levels of the inflammatory cascade, all of them share notable cytotoxicity and poor water solubility that limit their physiological bioavailability [16]. To overcome these limitations, the use of tailored nanocarriers for their encapsulation has gained great attention in recent years [17]. Particularly, polymer-based nanoparticles (NPs) are promising anti-inflammatory delivery systems to control drug release, prolong drug stability, reduce drug toxicity and increase drug bioavailability; and have been the object of numerous investigations [18].

In this sense, some advances on the encapsulation of anti-inflammatory drugs into polymeric nanovehicles have been made for OA therapy. In particular, considerable work trying to combine DEX with nanotechnology has been developed, since DEX possesses powerful anti-inflammatory activity, even at low doses [19]. It has been recently encapsulated alone [20,21,22], or in combination with other factors, such as other anti-inflammatory molecules like ketoprofen [23] or small interfering RNA [24], and covalently conjugated, for example, with the avidin protein [25] with positive effects both in vitro and in vivo. In the case of CLX, CLX poly(1-vinyl-2-pyrrolidone) solid dispersion NPs [26], or CLX-loaded hyaluronan NPs [27], were prepared to improve this drug bioavailability. In other work, CLX-loaded silk fibroin NPs showed anti-inflammatory and antioxidant activities by the in vitro reduction of NO, IL-6 and RANTES [28]. On the other hand, to the best of our knowledge, scarce literature on TNX-loaded polymeric NPs can be found for the treatment of arthritic diseases. Injectable formulations like in situ-forming microparticles, showed promising results as TNX delivery vehicles in terms of anti-inflammatory and antioxidant activity for rheumatoid arthritis [29]. Moreover, TNX has been encapsulated into delivery systems such as microemulsion-based formulations [30], proniosomes [31] and ultradeformable vesicles based on surfactant molecules [32], for transdermal and topical applications.

In this context, we hypothesized that the use of biocompatible amphiphilic core-shell NPs, which are able to entrap in their hydrophobic core anti-inflammatory drugs, could provide a nanocarrier platform to act in situ as immunomodulatory therapeutic systems of OA by regulating the cellular release of inflammatory factors. Antecedents of this type of nanocarrier by the authors have given promising results for their application in cancer, sensorineural hearing loss, inflammatory and oxidative stress related pathologies [33,34,35,36,37,38,39]. Thus, the aim of this work was the nanoencapsulation of three types of anti-inflammatory drugs (CLX, TNX and DEX) into core-shell terpolymer NPs consisting of vitamin E methacrylate, 1-vinyl-2-pyrrolidone and *N*-vinylcaprolactam (poly(MVE-*co*-VP-*co*-VC)), and the study of their physicochemical parameters and the cellular release of osteoarthritic inflammatory mediators. These nanoparticulated systems are proposed to be intra-articularly injected for the treatment of mild to moderate OA. Physicochemical characterization of the loaded NPs was performed in terms of morphological evaluation, size distribution, zeta potential and encapsulation efficiency. Stability of the NPs in the long term was followed up to seven months in static conditions. The in vitro cytotoxicity of the NPs was studied on human articular chondrocytes and murine RAW264.7 macrophages, while the anti-inflammatory potential was characterized in terms of modulation of the release of osteoarthritic inflammatory mediators (NO, TNF-α, IL-1β, IL-6, PGE_2_ and IL-10) by LPS-stimulated RAW264.7. Eventually, the in vivo biocompatibility was assessed by subcutaneously injecting the NPs in a rat model, followed by a histological evaluation.

## 2. Materials and Methods

### 2.1. Chemicals

α-tocopherol (Sigma-Aldrich, Saint Louis, MO, USA), methacryloyl chloride (Sigma-Aldrich, Saint Louis, MO, USA), triethylamine (Scharlau, Barcelona, Spain), tetrabutylammonium iodide (Sigma-Aldrich, Saint Louis, MO, USA), dichloromethane (Sigma-Aldrich, Saint Louis, MO, USA), hexane (Sigma-Aldrich, Saint Louis, MO, USA), hydrochloric acid (VWR, Rochester, NY, USA), sodium hydroxide (Sigma-Aldrich, Saint Louis, MO, USA), 1,4-dioxane (Panreac, Barcelona, Spain), anhydrous dioxane (Sigma-Aldrich, Saint Louis, MO, USA), *N*-vinylcaprolactam (VC, Sigma-Aldrich), 1-vinyl-2-pyrrolidone (VP, Sigma-Aldrich) and 2,2′-azobisisobutyronitrile (AIBN, Sigma-Aldrich) were used for the preparation of a terpolymer (poly(MVE-*co*-VP-*co*-VC)), as described in a previous work [40].

Celecoxib (CLX, Sigma-Aldrich), dexamethasone (DEX, Sigma-Aldrich), tenoxicam (TNX, Alfa Aesar, Kandel, Germany) and ethanol (VWR, Matsonford Road, Radnor, PA, USA) were used as received for the synthesis of the NPs. Sodium chloride (NaCl, Sigma-Aldrich, Saint Louis, MO, USA) and sodium phosphate dibasic (Na_2_HPO_4_, Sigma-Aldrich, Saint Louis, MO, USA) were used for the preparation of a phosphate buffered saline (PBS) solution in which NPs were synthesized and diluted.

### 2.2. Preparation and Physicochemical Characterization of NPs

NPs were prepared by the nanoprecipitation method [40]. Different initial drug concentrations (2–20% *w*/*w* with respect to the terpolymer) were used in order to optimize the NPs in terms of physicochemical and biological properties. Briefly, the terpolymer (50 mg mL^−1^) and the corresponding drug at different % *w*/*w* (5, 10, 20 for CLX; 2, 5, 10 for TNX; and 10, 15, 20 for DEX) were dissolved in dioxane and added dropwise over PBS under constant stirring. Unloaded NPs were prepared using the same methodology as a blank for the encapsulation efficiency studies. NP suspensions were obtained at a final concentration of 2 mg mL^−1^ and were named according to the initial drug content (CLX-5, CLX-10, CLX-20, TNX-2, TNX-5, TNX-10, DEX-10, DEX-15, DEX-20). Suspensions were purified by dialysis against PBS for 72 h to eliminate the dioxane and the nonencapsulated drug, and finally stored at 4 °C. Physicochemical and biological characterization of unloaded NPs was not studied because it was assessed in a previous work [40].

NP mean hydrodynamic diameter (D_h_), size distribution and polydispersity index (PDI) were determined by Dynamic Light Scattering (DLS) while zeta potential (ξ) was measured by Laser Doppler Electrophoresis (LDE) using a Zetasizer Nano ZS (Malvern Instruments) at 25 °C. Experiments were performed using a NP concentration of 0.50 mg mL^−1^. NP stability was investigated by assessing the D_h_ and the size distribution of the NPs when stored under static conditions for seven months at 4 °C at different time points. Experiments were performed in triplicate and results were expressed as mean value ± standard deviation (SD). Drug encapsulation efficiency (EE) was studied by ultraviolet (UV) spectroscopy. First, NP suspensions were freeze-dried and dissolved in ethanol for 24 h. Samples were then centrifuged at 10,000 rpm and supernatants containing the drugs were analyzed at the UV absorption maxima of the corresponding drug (i.e., 255, 355 and 238 nm for CLX, TNX and DEX, respectively) with a Nanodrop One^c^ Microvolume UV-Vis Spectrophotometer (Thermo Scientific^TM^). Unloaded NPs were used as a blank, whose absorbance value was subtracted to the one of loaded NPs. EE (%) was then calculated as (experimental drug/initial drug)x100 being the experimentally detected and the initial drug concentrations, respectively. Experiments were performed in triplicate for each formulation and expressed as mean value ± SD. Scanning electron microscopy (SEM) was used for the morphological characterization of the NPs at a NP concentration of 0.04 mg mL^−1^ using a Hitachi SU8000 TED, cold-emission field emission SEM microscope working at an accelerating voltage 30 kV.

### 2.3. Cell Cultures and Biological Products

High glucose Dulbecco’s Modified Eagle’s Medium (DMEM, D6546, Sigma-Aldrich, Saint Louis, MO, USA); high glucose, HEPES, no phenol red DMEM (Gibco, 2106329, Thermo Fisher, UK), AlamarBlue^®^ (Invitrogen, Eugene, OR, USA), Trypsin-EDTA solution (Sigma-Aldrich, Saint Louis MO, USA), Trypan Blue (Sigma-Aldrich, Saint Louis, MO, USA), Dulbecco’s Phosphate Buffered Saline (PBS, Merck, UK), Griess reagent (Sigma-Aldrich, Saint Louis, MO, USA) and lipopolysaccharide from *Escherichia coli* (LPS, Sigma-Aldrich) were used for the cellular assays.

NP toxicity was assessed using human articular chondrocytes (HC-a, Innoprot, P10970, Bizkaia, Spain) and a murine macrophage cell line (RAW264.7, Sigma-Aldrich, 91062702, UK). RAW264.7 was also used for the NO quantification, the mouse inflammation antibody array and TNF-α, IL-1β, IL-6, PGE_2_ and IL-10 ELISA kits. HC-a were grown and maintained using a chondrocyte medium kit (Innoprot, P60137, Bizkaia, Spain) while RAW264.7 were grown and maintained in high glucose DMEM supplemented with 10% Fetal Bovine Serum (FBS, Gibco, Brazil), 2% L-glutamine (Sigma-Aldrich, Saint Louis, MO, USA) and 1% penicillin/streptomycin (Sigma-Aldrich, Saint Louis, MO, USA), both at 37°C in a humidified atmosphere of 5% CO_2_. When reaching 80% confluence, HC-a were detached with trypsin-EDTA and RAW264.7 by scraping. NP suspensions were sterilized by filtering through 0.22 µm polyethersulfone membranes (Millex-GP PES Millipore Express, Sigma-Aldrich, Darmstadt, Germany) and diluted with PBS to obtain different NP concentrations (0.06, 0.12, 0.25, 0.50, 1.00 mg mL^−1^).

### 2.4. NP Cytotoxicity

NP toxicity was investigated on HC-a at different periods of time (24 and 48 h, 7 and 14 days). 2 × 10^4^ cells/well were seeded into 24 well culture plates and incubated for 24 h. The culture medium was then replaced with a fresh one and with the NPs (1:1). After each time (24 and 48 h, 7 and 14 days) NPs were removed, cells were washed with PBS, treated with a 10% AlamarBlue^®^ solution in phenol red-free DMEM and incubated for 3 h. After this time, fluorescence was quantified at an excitation/emission of 590/530 nm using a fluorescence microplate reader (Biotek Synergy HT spectrophotometer, BioTek Instruments, Winooski, VT, USA). For 48 h, 7 and 14 day assays, NPs and culture medium were replaced each two days. For each time, cells treated with PBS were used as control (CNT). Experiments were performed using eight replicates per formulation and results were expressed as mean ± SD. Analysis of variance (ANOVA) was performed at a significance level of *p* < 0.05.

Cytotoxicity of free drugs on HC-a was also investigated after 24 h and compared to nanoencapsulated drugs in the different NP systems at the different NP concentrations. Due to the low solubility of these drugs in aqueous media, a mother solution of each drug was prepared in DMSO. Serial dilutions were prepared using HC-a culture medium, maintaining the final DMSO concentration lower than 1% *v/v* in the cell culture experiments. Cells were seeded at 2 × 10^4^ cells/well into 24 well culture plates and incubated for 24 h. The medium was then replaced by the serial drug solutions and incubated for additional 24 h. Then, the medium was removed, cells were washed with PBS and treated with a 10% AlamarBlue^®^ solution. After 3 h of incubation, fluorescence was monitored. Cells treated with the HC-a culture medium were used as the 100% viability control. Eight replicates were used for each sample.

The cytotoxicity of the NPs was also studied in RAW264.7 after 24 h. In this case, cells were seeded at 2 × 10^4^ cells/well into 96 well culture plates and incubated for 24 h. The medium was then replaced with a fresh one and with the NPs (1:1) for 24 h. Finally, the same AlamarBlue^®^ protocol used for HC-a was followed. Cells treated with PBS were used as control (CNT). Experiments were performed using eight replicates per formulation and results were expressed as mean ± SD. ANOVA was performed at a significance level of *p* < 0.05.

Cytotoxicity of free drugs on RAW264.7 was also investigated after 24 h. A mother solution of each drug was prepared in DMSO and serial dilutions were prepared with RAW264.7 culture medium. Cells were seeded at 2 × 10^4^ cells/well into 96 well culture plates and incubated for 24 h. The medium was replaced by the serial drug solutions and incubated for an additional 24 h. Then, the medium was removed, cells were washed with PBS and treated with a 10% AlamarBlue^®^ solution. After 3 h of incubation, fluorescence was monitored. Cell viability of RAW264.7 treated with culture medium was taken as 100%. Eight replicates were used for each sample.

### 2.5. Quantification of NO Cellular Release

NO quantification in LPS-stimulated RAW264.7 was used as an in vitro inflammation model. RAW264.7 were cultured in 96 well culture plates at 2 × 10^4^ cells/well and incubated for 24 h, then, the medium was replaced by fresh one containing LPS (1 μg mL^−1^) and the NP suspensions (1:1). After 24 h, supernatants were collected, and NO was quantified using the Griess method by reacting the extracts with the Griess solution (1:1). After 15 min of reaction, absorbance was detected using a microplate reader at 540 nm. The concentration of NO was obtained using a sodium nitrite serial dilution curve. After the collection of the extracts, an AlamarBlue^®^ assay was performed to the cells following the protocol described in 2.4 for data normalization. LPS-stimulated cells treated with PBS were used as control (LPS+). NO basal levels of unstimulated cells were also studied (LPS-). Experiments were performed using eight replicates per formulation and results were expressed as mean ± SD. ANOVA was performed at significance levels of *p* < 0.05, *p* < 0.005 and *p* < 0.001.

### 2.6. Semi-Quantitative Inflammation Antibody Array

A mouse inflammation antibody array (ab133999, abcam, Spain) with 40 inflammatory targets was used to semi-quantitatively study the anti-inflammatory effect of the NPs. RAW264.7 were stimulated with LPS (500 ng mL^−1^) in culture medium without FBS to simulate inflammatory conditions. Five membranes were used for this experiment: a control membrane (CNT) was exposed to RAW264.7 culture medium without FBS; an inflammatory membrane (LPS) was exposed to supernatants of LPS-stimulated cells exposed to PBS and three membranes were exposed to the cellular supernatants of LPS-stimulated cells treated with the corresponding NPs at 0.50 mg mL^−1^ (CLX-10, TNX-5 or DEX-15). RAW264.7 were seeded at 3 × 10^5^ cells/well into six well plates. After 24 h of incubation, the culture medium was replaced with 1 mL of NPs and 1 mL of fresh culture medium without FBS containing LPS (500 ng mL^−1^). For the LPS membrane, the medium was replaced with 1 mL of culture medium without FBS containing LPS (500 ng mL^−1^) and 1 mL of PBS. Finally, supernatants were taken after 24 h of exposure to NPs and stored at −20 °C until use. Membrane chemiluminescence was detected and quantified using a ChemiDoc™ XRS. Two replicates for each inflammatory mediator were studied per membrane. Results are expressed as mean ± SD and values are relative to the Positive control of each membrane, which is given an arbitrary value of 1. ANOVA was performed at significance levels of *p* < 0.05, *p* < 0.005 and *p* < 0.001.

### 2.7. Quantification of TNF-α, IL-1β, IL-6, PGE_2_ and IL-10 Cellular Release

NP effect on the release of five inflammatory mediators in LPS+ RAW264.7 was further quantified using mouse TNF-α, IL-1β and IL-10 ELISA kits purchased from Sigma-Aldrich (RAB0477, RAB0274 and RAB0245); and mouse IL-6 and PGE_2_ ELISA kits purchased from abcam (ab222503 and ab133021). In brief, cells were seeded into six well culture plates at a density of 3 × 10^5^ cells/well and cultured for 24 h, then, cells were activated with LPS in RAW264.7 culture medium (1 µg mL^−1^ for IL-1β quantification and 500 ng mL^−1^ for TNF-α, IL-6, PGE_2_ and IL-10) and exposed simultaneously to NPs (CLX-10, TNX-5 and DEX-15 at 0.50 mg mL^−1^). Following a 24 h incubation, supernatants were collected and stored at −20 °C until use. Levels of TNF-α, IL-1β, IL-6, PGE_2_ and IL-10 in cell culture supernatants were determined by the corresponding ELISA kit according to the protocol recommended by the manufacturer. LPS-stimulated and unstimulated cells treated with PBS were used as controls (LPS+ and LPS-, respectively). Experiments were performed using five replicates per formulation and results were expressed as mean ± SD. ANOVA were performed comparing tested NPs and LPS+ at significance levels of *p* < 0.05, *p* < 0.005 and *p* < 0.001 and comparing NPs with each other at significance levels of *p* < 0.05, *p* < 0.005 and *p* < 0.001.

### 2.8. In Vivo Biocompatibility Evaluation

#### 2.8.1. Animals

Animal experiments were carried out according to the European Directive (2010/63/EU) and the National Spanish Law (RD 53/2013). The Ethical Committee of University of Salamanca approved the surgical protocols (register number: 035).

#### 2.8.2. In Vivo Subcutaneous Injection of NPs

Animals were acclimatized for at least twoweeks prior to surgery. Ten Wistar rats were anesthetized with 1.5% isofluorane (Vetflurane^®^), shaved with an electric shaver and sterilized with an antiseptic solution (povidone-iodine, Betadine^®^). Four dorsal subcutaneous injections of PBS (control) or NPs (CLX-10, TNX-5 and DEX-15, 0.50 mg mL^−1^) using 21G needles and a volume of 1 mL, were performed in each rat. Animals were euthanized by anesthetic overdose at one and two weeks post injection. Rat backs were shaved over the injection site and tissues were harvested for histological evaluation.

#### 2.8.3. Histological Study

To evaluate the inflammatory reaction to NPs, tissue fragments around the injection site were fixed with 10% formalin solution and embedded in paraffin. Samples were sectioned (5 µm) along the longitudinal axis of the skin and stained with hematoxylin and eosin (H-E). The images were obtained with a light microscope (Nikon Eclipse 90i) coupled to a Nikon Digital Sight DS-smc camera (Nikon Corporation, Tokyo, Japan).

## 3. Results and Discussion

Nonsteroidal anti-inflammatory drugs (NSAIDs) (both traditional or specific cyclooxygenase-2, COX-2, inhibitors) and glucocorticoids, are the keystone of the treatment of inflammation and pain related to osteoarthritis (OA). NSAIDs are normally prescribed for oral administration or as topical agents when patients present gastrointestinal problems, while glucocorticoids can be taken orally for mild stages of OA or intra-articularly administered for patients with more severe OA symptoms [9]. In the present study, tenoxicam (TNX) as a traditional NSAID, celecoxib (CLX) as a selective COX-2 inhibitor and dexamethasone (DEX) as a glucocorticoid, were selected for studying their anti-inflammatory potential after their nanoencapsulation into polymeric nanoparticles (NPs) based on an amphiphilic terpolymer, in order to reduce drug cytotoxicity and improve drug stability in aqueous media. Some relevant properties of the three drugs are summarized in Table 1.

### 3.1. Preparation and Physicochemical Characterization of NPs

**Figure 1:**
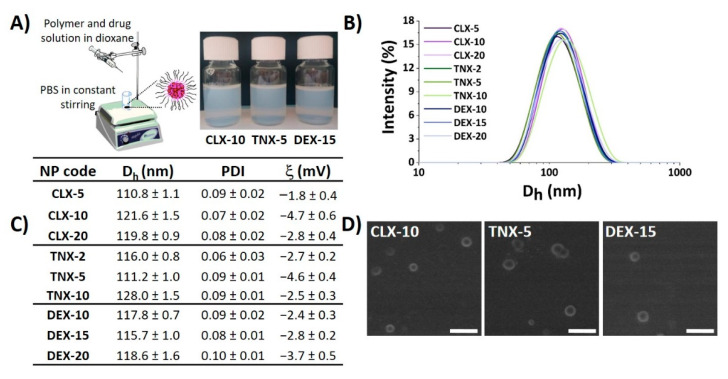
Physicochemical properties of CLX, TNX and DEX-loaded NPs. (**A**) NP suspensions obtained by the nanoprecipitation method. Image created using ChemDraw. (**B**) NP size distribution immediately after synthesis. (**C**) Hydrodynamic diameter (D_h_), polydispersity index (PDI) and zeta potential (ξ) values of NPs. (**D**) SEM micrographs of CLX-10, TNX-5 and DEX-15 NPs. Scale bar: 300 nm.

Nanoprecipitation is a simple, cost-effective technique widely used for the encapsulation of poorly-water soluble drugs [44,45,46]. Here, CLX, TNX or DEX-loaded polymeric NPs were obtained by the nanoprecipitation method using the terpolymer poly(MVE-*co*-VP-*co*-VC) (Figure 1A). A scheme of the synthesis of poly(MVE-*co*-VP-*co*-VC) showing its chemical structure, its ^1^H-NMR spectrum and its main physicochemical parameters is presented in Figure S1. The appropriate hydrophilic-hydrophobic balance of the polymer allows its self-assembly during nanoprecipitation, giving rise to NPs entrapping the drug into the hydrophobic core [40]. In all cases, NPs showed narrow unimodal size distributions by light scattering, as shown in Figure 1B, with polydispersity index (PDI) values below 0.1, demonstrating good size homogeneity in all systems independently of the encapsulated drug. Mean hydrodynamic diameter (D_h_), PDI and zeta potential (ξ) of NPs are given in Figure 1C. Hydrodynamic diameters ranged between 110 and 130 nm. Based on the literature, particle diameters below 200 nm are appropriate for particle cellular internalization, while NPs with diameters higher than 100 nm are capable of avoiding their elimination by the reticuloendothelial system [47], making this range of sizes (100–200 nm) particularly suitable for nanocarriers used in biological applications.

NP aggregation is one of the key problems of this type of drug delivery system. Consequently, stability of the NPs in terms of aggregation was evaluated when stored under static conditions at 4 °C during seven months. As indicated by Dynamic Light Scattering (DLS) (Figure S2), after seven months all NPs maintained their size below 140 nm, PDI values below 0.2 and unimodal size distributions, suggesting the stability of the NPs and minimal aggregation.

Zeta potentials were slightly negative, ranging between −1 and −5 mV, as observed in Figure 1C, due to the presence of 1-vinyl-2-pyrrolidone (VP) and *N*-vinylcaprolactam (VC)-rich domains in the shell of the NPs [37]. It is widely reported that cationic and neutrally charged NPs show the highest transport efficiency compared to negatively charged ones due to the charge attraction between positive NPs and negative cell membrane surfaces, thereby increasing the rate and extent of particle internalization [48]. Scanning electron microscopy (SEM) images, presented in Figure 1D, showed spherical particles for the three types of loaded NPs, morphology that has demonstrated to facilitate NP internalization compared to other like cubic or rod morphologies [49,50]. In addition, the average diameter and size distribution homogeneity agreed with the hydrodynamic light scattering studies.

NP physicochemical properties including size, surface charge, surface chemistry or morphology, among others, are important factors in order to be sequestered by cells, especially inflammatory cells like macrophages [51]. In general, spherical, positively charged or neutral charged NPs having sizes between 100–200 nm, are thought to be optimal systems to be cell-sequestered. Thus, the hydrodynamic properties of the NPs prepared here are ideal for an efficient cellular uptake.

Polymer composition and an appropriate hydrophilic-hydrophobic balance of amphiphilic polymers are critical parameters in drug encapsulation efficiency. In this sense, Thayumanavan et al. discovered that varying the combination between random and block copolymers in polymeric coassemblies leads to the possibility of modulating hydrophobic cargo loading capacity and cargo release behavior [52]. In the investigations of Therasima and coworkers, controlling the primary polymer structure in terms of composition and chain length could tune thermoresponsive nanomicelle size, aggregation number and cloud points [53]. Here, the amphiphilic structure of poly(MVE-*co*-VP-*co*-VC) (see Figure S1) allowed the encapsulation of three hydrophobic drugs. Drug hydrophilicity was also critical for its encapsulation since the higher the water solubility, the lower the encapsulation efficiency achieved. As seen in Table 1, DEX is the most water soluble drug, followed by TNX and CLX. Furthermore, drug logP, which is the partition coefficient of a molecule between aqueous and lipophilic phases, is higher for the most hydrophobic drug (CLX) followed by TNX and DEX (see Table 1). In the case of the encapsulation efficiency, the highest values were obtained for CLX followed by TNX and DEX (Table 2), in agreement with logP values and opposing water solubility values. This core-shell nanovehicle is formed by a core of hydrophobic MVE rich moieties and a shell or corona of hydrophilic rich moieties, based mainly on VP [40]. MVE-based hydrophobic blocks forming the inner core entrapped the drug by hydrophobic interactions and hydrogen bonding. The higher the hydrophobicity of the drug, the greater the affinity between the core and the drug and, as a consequence, the greater the amount of entrapped drug. Besides, for CLX NPs the encapsulation efficiency was also dependent of the feed drug concentration, the encapsulation efficiency (EE) being higher (72%) for the lower initial drug amount (2% *w*/*w*). For TNX NPs, EE was similar for the three formulations, while for DEX NPs the highest value was reached for the lowest feed drug concentration assayed (10% *w*/*w*), as in the case of CLX NPs. Encapsulated drug concentrations are also shown in Table 2. As can be ascertained, for CLX NPs and TNX NPs encapsulated drug concentrations increased with feed concentration, reaching the highest value for CLX-20 (0.156 mg mL^−1^). In the case of DEX NPs, very similar values, around 0.050 mg mL^−1^, were achieved. These encapsulated drug concentrations determine the bioactivity of the NPs in terms of cytotoxicity and nitric oxide (NO) reduction, as will be explained in the following sections.

Comparing the hydrodynamic properties and the EE acquired here to those of previously described curcumin (CUR)-loaded NPs, very similar results were achieved regarding diameters (114–135 nm for CUR NPs) and zeta potentials (around −4 mV for CUR NPs) [40]. However, EE were higher in the case of CUR, with values between 72–79% due to the lower water solubility of this drug (3.12 mg mL^−1^) [54] in comparison to CLX, TNX and DEX, which may allow more and stronger interactions of CUR with the particle core.

Nanoprecipitation has been recently used for the encapsulation of these drugs into different polymeric nanovehicles, obtaining systems with different physicochemical properties. In the case of CLX, higher encapsulation efficiencies (around 90%) were obtained in the CLX-loaded hyaluronan nanocapsules of El-Gogary et al. [27] or in the propylene glycol alginate sodium sulfate based pH-sensitive nanotherapeutic systems of Zhang et al. [55]. However, highly negatively charged particles were obtained in both cases, below −26 mV, with bigger particle sizes (>129 nm) and PDI values (>0.1).

The same happened with recently described DEX-loaded NPs, in which encapsulation efficiencies were around 50% for the DEX-loaded PLGA-PEG NPs of Albisa et al. [56], although sizes between 250–400 nm were obtained. In the case of Chiesa et al. [57] smaller NPs (150 nm) entrapping DEX and using a dodecapeptide (GE11)-PLGA based conjugate were fabricated, although NPs presented highly negative surface charges (−25 mV). DEX has also been nanoencapsulated, for instance, into a mixture of two pseudoblock polymer drugs, poly(VP-*co*-MVE) and poly(VI-*co*-HEI), VI being 1-vinylimidazole and HEI being a methacrylic derivative of ibuprofen, with NP sizes of 179–211 nm, surface charges of −2.6 to −0.5 and encapsulation efficiencies of 36–59% [38]. These results were closer to those reached here due to the presence of the copolymer system poly(VP-*co*-MVE) with a similar composition to the terpolymer poly(MVE-*co*-VP-*co*-VC) used in this work.

In the case of TNX, no polymeric nanocarriers prepared by nanoprecipitation have been found. All in all, these CLX and DEX-loaded NPs have, in general, smaller hydrodynamic diameters than those reported in recent polymeric NPs prepared by nanoprecipitation, and surface charges nearer neutral, due to the presence of vinyl groups at the NP shell, making this nanoparticulated system appropriate for biological applications. Furthermore, with similar characteristics compared to these systems, previously described terpolymer NPs encapsulating CUR demonstrated to be successfully endocyted by human articular chondrocytes (HC-a) and murine macrophages (RAW264.7) [40]. Moreover, encapsulation efficiencies were high enough to observe in vitro biological effects, as explained hereafter.

### 3.2. Effect on HC-a Viability

**Figure 2:**
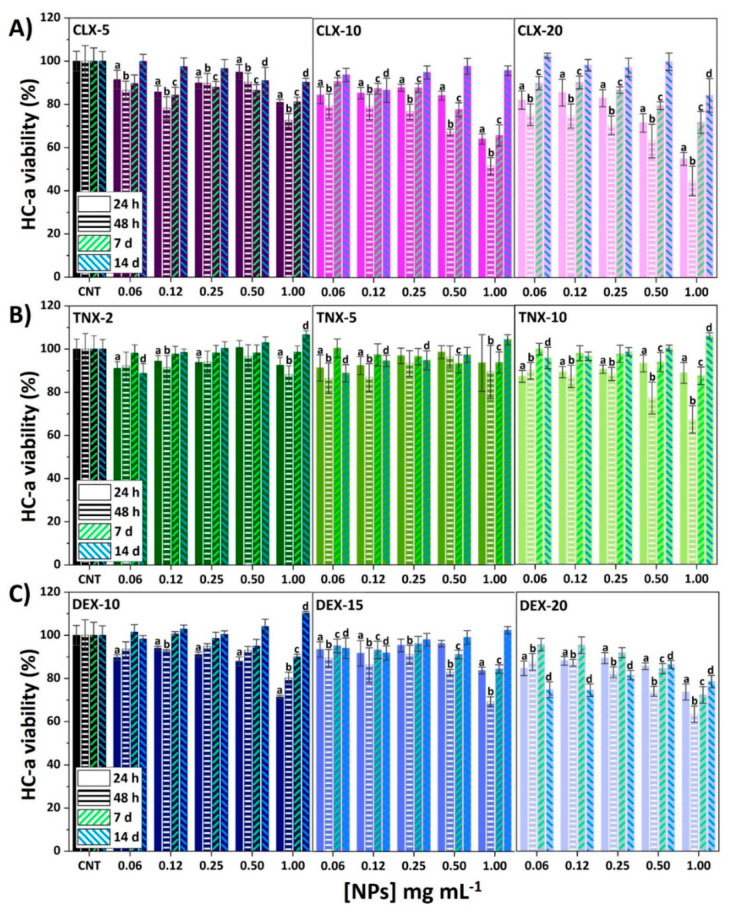
Effect of (**A**) CLX, (**B**) TNX and (**C**) DEX NPs on HC-a viability after 24 and 48 h, 7 and 14 days. Mean ± SD values are relative to control cells without NP treatment (CNT) for each time, in which cell viability was taken as 100%. ANOVA of the results was performed with respect to their corresponding CNT at a significance level of *p* < 0.05 (a, b, c and d correspond to statistical significance for 24 h, 48 h, 7 and 14 days samples, respectively).

One of the main drawbacks of intra-articularly injecting free anti-inflammatory drugs is their high toxicity when in contact with cartilage, and the formation of crystals and further cartilage damage due to the crystalline nature of some of them [9]. Therefore, in order to assess possible toxic effects of these drug-loaded NPs, an AlamarBlue^®^ test was carried out on HC-a at different time periods (24 and 48 h, 7 and 14 days). Results of HC-a viability are represented in Figure 2 for all tested NPs.

Cytotoxicity of CLX NPs was dependent on time, CLX concentration and NP concentration (Figure 2A). The lowest HC-a viability was observed after 48 h of NP treatment for all CLX formulations and, after this time, cell viability was recovered reaching values over 80% for all the formulations after 14 days. CLX-5 NPs did not affect cell viability at any NP concentration, while for CLX-10 and CLX-20, viabilities below 70% were observed for the two most concentrated suspensions (0.50 and 1.00 mg mL^−1^). Figure S3 shows HC-a viabilities after 24 h in contact with free or nanoencapsulated drugs against drug concentration. For CLX (Figure S3A), the three systems CLX-5, CLX-10 and CLX-20 achieved a remarkable reduction of free CLX cytotoxicity. Hence, going back to Figure 2A, a wide range of CLX and NP concentrations were demonstrated to be noncytotoxic at any time, reaching only cytotoxic effects for CLX encapsulated concentrations of 0.025 and 0.050 mg mL^−1^ (CLX-10 at 0.50 and 1.00 mg mL^−1^ of NPs) and 0.039 and 0.078 mg mL^−1^ (CLX-20 at 0.50 and 1.00 mg mL^−1^ of NPs). In any case, the three formulations presented lower toxicity than free CLX at the same concentration as the encapsulated drug.

Regarding TNX NPs (Figure 2B), the only NP suspension reducing HC-a viability below 70% was TNX-10 at 1.00 mg mL^−1^ after 48 h. However, an interesting increase in cell viability after 14 days was observed for TNX-2, 5 and 10 NPs at 1.00 mg mL^−1^ that was statistically significant (*p* < 0.05) for TNX-2 and TNX-10. Comparing the results at 24 h with the results of free TNX (Figure S3B) no statistically significant changes were observed between nanoencapsulated or free TNX, as the concentrations of nanoencapsulated TNX were not cytotoxic in its free form.

For DEX NPs (Figure 2C), the lowest viabilities were found after 24 or 48 h, having viabilities below 70% for DEX-10, 15 and 20 at 1.00 mg mL^−1^. Moreover, the same effect as for TNX was observed for DEX-10 at 1.00 mg mL^−1^ after 14 days, in which a significant increase on cell viability was observed. In any case, after 14 days of NP treatment, HC-a presented viabilities over 80% for all NPs. As observed in Figure S3C, the nanoencapsulation of DEX led to a significant reduction of its free form cytotoxicity, confirming the suitability of this polymeric system to reduce the cytotoxicity of both, CLX and DEX.

In conclusion, a reduction of CLX and DEX toxicity in HC-a was observed after their nanoencapsulation. Additionally, a wide range of CLX, TNX and DEX formulations did not cause cytotoxic effects on HC-a, making them suitable for the treatment of OA or other cartilage-related conditions.

### 3.3. Effect on RAW264.7 Viability and NO Release

**Figure 3:**
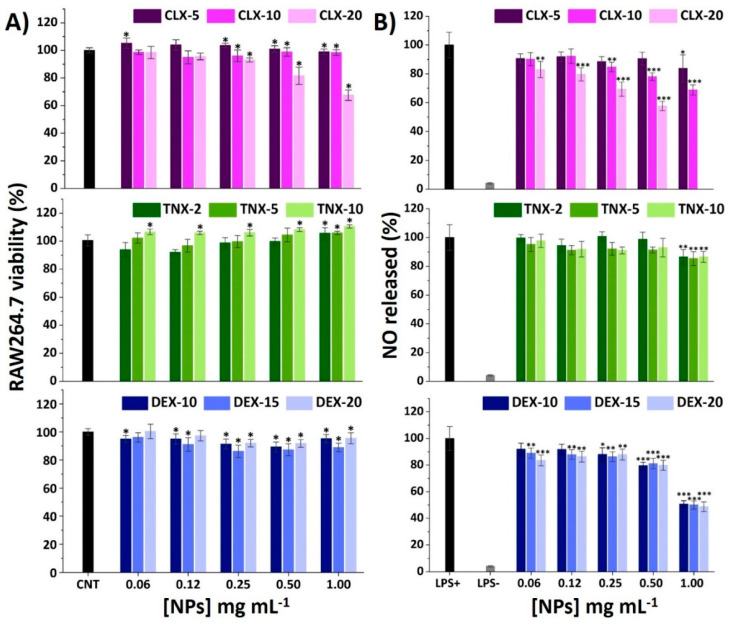
(**A**) Effect of CLX, TNX and DEX NPs on RAW264.7 viability after 24 h. Mean ± SD values are relative to control cells without NP treatment (CNT), in which cell viability was taken as 100%. ANOVA of the results was performed with respect to CNT at a significance level of * *p* < 0.05. (**B**) Effect of CLX, TNX and DEX NPs on NO production in LPS-stimulated cells. Mean ± SD values are relative to control LPS+, in which NO production was taken as 100%. CLX-20 NPs at a concentration of 1.00 mg mL^−1^ were not tested since they showed cytotoxic effects on the AlamarBlue^®^ viability test. ANOVA of the results was performed with respect to LPS+ at significance levels of * *p* < 0.05, ** *p* < 0.005 and *** *p* < 0.001.

Macrophages are key regulators of OA-related inflammation, secreting inflammatory mediators such as cytokines and chemokines, controlling the activity of the adaptive immune system, and also conditioning other cells such as chondrocytes [58,59,60]. To elucidate the effect of the NPs on the viability of RAW264.7 macrophages, an AlamarBlue^®^ assay was performed after 24 h of exposure to NPs using the same protocol as for HC-a. Results are shown in Figure 3A. For CLX NPs, the only formulation causing a decrease on viability below 70% was CLX-20 at 1.00 mg mL^−1^. This formulation presents the highest drug content as can be seen in Table 2 (0.078 in 1.00 mg mL^−1^ NP suspension). For the rest of NPs, cell viabilities were maintained between 80 and 110%. Comparing these results with the ones of free CLX (Figure S4A) a reduction on the cytotoxicity of CLX when encapsulated into this polymeric vehicle was observed.

When treated with TNX NPs, RAW264.7 viability increased with TNX load and NP concentration, reaching 111% of viability for TNX-10 at 1.00 mg mL^−1^, as happened to HC-a (Figure 2B). Moreover, nonencapsulated TNX (free form) did not cause any cytotoxic effect at the TNX nanoencapsulated concentrations, as occurred with HC-a, so the comparison of free and nanoencapsulated TNX cytotoxicity could not be performed.

Finally, the treatment with DEX NPs resulted in cell viabilities around 100% in the whole range of the studied concentrations (0.06–1.00 mg mL^−1^). Comparing these results with the RAW264.7 viability results of free DEX (Figure S4C), a reduction of the drug cytotoxicity was achieved for similar concentrations of the free and nanoencapsulated drug, similar to CLX NPs. Therefore, the reduction in cytotoxicity obtained for CLX and DEX NPs in RAW264.7 further encourages the use of this polymeric nanocarrier as a delivery system to reduce the cytotoxic effects of these drugs.

It is well recognized that bacterial lipopolysaccharide (LPS) stimulation induces RAW264.7 M_0_ polarization into an M_1_ proinflammatory phenotype through the binding of LPS to cellular Toll-like receptors (TLR), particularly TLR4 [61,62]. This leads to the activation of the NF-κB signaling pathway activating the release of different proinflammatory mediators, such as nitric oxide (NO), reactive oxygen species (ROS) and different cytokines and chemokines. Among these factors, NO overproduction plays a major regulatory role in tissue damage associated with chronic inflammation presented in OA. Different polymeric NPs have demonstrated a reduction of NO in LPS-stimulated RAW264.7 encapsulating CLX [28] and DEX [23,63,64]. Unloaded NPs were deeply studied in a previous work and the reduction in NO production was not observed in LPS-stimulated RAW264.7 macrophages [40]. Hence, the immunomodulatory effects that are noticed here are expected to be due to the loaded anti-inflammatory drugs. Results of NO production for the studied NPs are shown in Figure 3B. Basal levels of NO in unstimulated cells treated with PBS (LPS-) were around 5% with respect to the LPS+ control (LPS-stimulated cells treated with PBS).

For CLX NPs, there was a drug content and NP concentration-dependent effect, reducing e NO release to 60% for CLX-20 at 0.50 mg mL^−1^ and to 70% for CLX-10 at 1.00 mg mL^−1^. In the case of TNX NPs, only the most concentrated NP suspensions (1.00 mg mL^−1^) significantly reduced NO levels to 85%, independently of TNX content. Ultimately, DEX NPs showed a NO reduction of 50% at 1.00 mg mL^−1^ for all drug contents without differences between them, which is in accordance with the very closed encapsulated DEX concentration values of these three systems (Table 2), suggesting that the anti-inflammatory effect of the NPs correlates with the amount of encapsulated drug.

These results suggest that the incorporation of the drugs inside the polymeric nanovehicle does not compromise their anti-inflammatory properties in terms of NO reduction. According to these results, CLX-10, TNX-5 and DEX-15 at 0.50 mg mL^−1^ were chosen for the following inflammatory studies.

### 3.4. Effect on RAW264.7 Release of Inflammatory Factors

**Figure 4:**
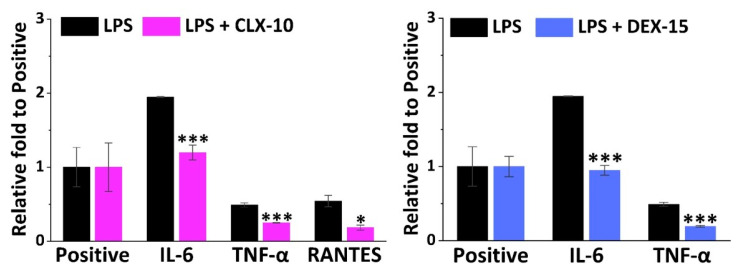
Effect of CLX-10 and DEX-15 NPs (0.50 mg mL^−1^) on the production of inflammatory mediators that were upregulated in LPS-stimulated RAW264.7 (LPS) using a mouse inflammation antibody array. Mean ± SD values are relative to the positive control of each membrane, which was given an arbitrary value of 1. ANOVA between inflammatory (LPS) and treatment membranes (LPS + CLX-10 and DEX-15) was performed at significance levels of * *p* < 0.05 and *** *p* < 0.001.

Apart from NO, M_1_ macrophages are in charge of the release of multiple other inflammatory mediators such as cytokines and chemokines that lead to leukocyte recruitment. To have a general view of the anti-inflammatory potential of the selected CLX-10, TNX-5 and DEX-15 NPs, a semi-quantitative inflammation antibody array was used. All the inflammatory mediators studied in the inflammation antibody array are listed in Table S1. Inflammatory factors that were released by RAW264.7 when stimulated with LPS were IL-1α, IL-4, IL-6, MCP-1, MIP-1α, MIP-1ϒ, TIMP-2, TNF-α, sTNF-RI, sTNF-RII, RANTES and MCSF. In Figure 4, the relative fold of those factors that were significantly repressed in LPS-stimulated cells by the action of CLX-10 and DEX-15 NPs are represented. TNX-5 NPs did not significantly reduce any of the overexpressed inflammatory factors in the inflammation antibody array.

IL-6 was the cytokine more notably overexpressed in LPS-stimulated RAW264.7, 1.9-fold to the positive control, and was significantly reduced in CLX-10 and DEX-15 NPs. TNF-α was the second most overproduced cytokine (0.5-fold to positive) and then reduced upon CLX-10 and DEX-5 NP treatment. For CLX-10, an additional chemokine, RANTES (regulated on activation, normal T cell expressed and secreted) was also reduced by the action of the NPs. The rest of the overproduced inflammatory factors were not significantly inhibited upon treatment with NPs.

In order to deepen the effect of the NPs in RAW264.7 secretion of osteoarthritic inflammatory factors, specific ELISAs were performed for TNF-α and IL-6 cytokines, but additionally for IL-1β, PGE_2_ and IL-10. Proinflammatory cytokines TNF-α, IL-1β and IL-6, and the inflammatory mediator PGE_2,_ have a key role in the development and progression of OA, which makes their inhibition an appealing potential target in the treatment of OA [8,65]. Anti-inflammatory cytokines such as IL-10 also play a major role in the pathophysiology of OA, counteracting the action of proinflammatory mediators.

**Figure 5:**
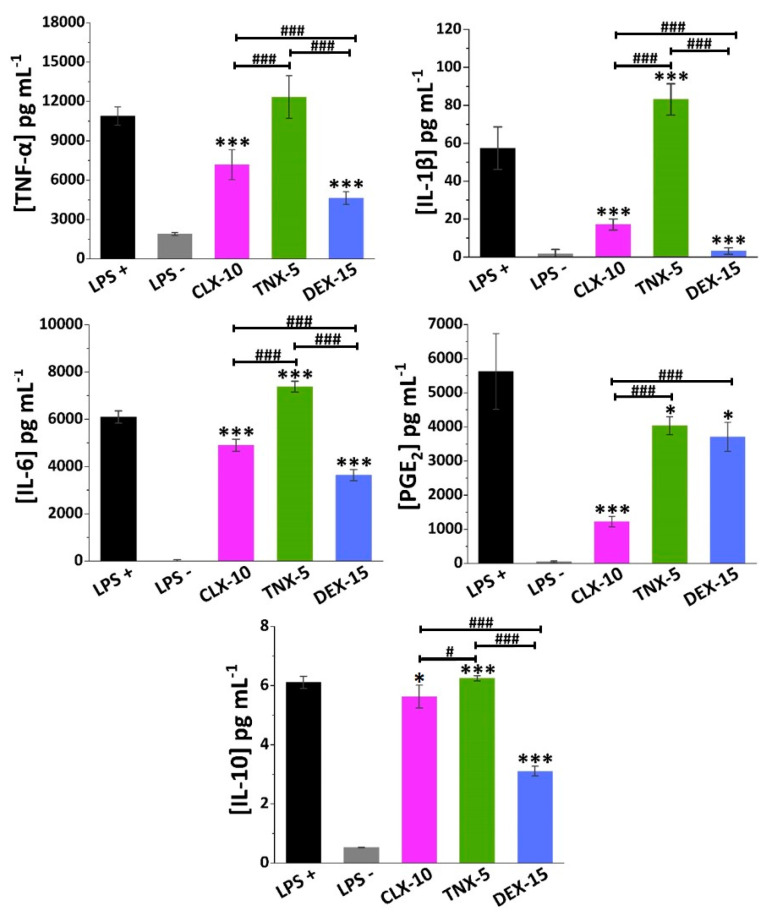
Effect of CLX-10, TNX-5 and DEX-15 (0.50 mg mL^−1^) on the release of the inflammatory mediators TNF-α, IL-1β, IL-6, PGE_2_ and IL-10 in LPS+ RAW264.7 using ELISA kits. Data are represented as mean ± SD values. ANOVA between each NP formulation and LPS+ (* *p* < 0.05 and *** *p* < 0.001) and between NPs (^#^ *p* < 0.05 and ^###^ *p* < 0.001) were performed.

In LPS-stimulated (LPS+) RAW264.7 TNF-α, IL-6 and PGE_2_ were produced to a greater extent (11,000, 6000 and 5500 pg mL^−1^, respectively) compared to IL-1β and IL-10 (58 and 6 pg mL^−1^, respectively), as seen in Figure 5.

NSAIDs exert their anti-inflammatory action mainly through the unspecific inhibition of both COX-1 and COX-2 enzymes, in the case of traditional NSAIDs, and selectively COX-2, in the case of coxibs. These COX enzymes are in charge of prostanoids synthesis from arachidonic acid, including PGE_2_, one of the major catabolic mediators involved in cartilage inflammation and degradation during OA [66]. However, while COX-1 regulates many cellular processes related, for instance, to the gastrointestinal and renal tracts, COX-2 is an inducible enzyme that increases during inflammatory processes. Nevertheless, other anti-inflammatory COX-independent pathways have been reported for NSAIDs, including their interaction with transcription factors like NF-κB or AP-1, or with cellular kinases that regulate gene expression of inflammatory molecules like NO, COX-2, TNF-α, IL-1β or IL-6, among others [67,68].

TNX is a traditional NSAID of the oxicam family, acting as an inhibitor of both COX enzymes, but primarily COX-1. Here, TNX-5 NPs reduced PGE_2_ RAW264.7 release while increasing IL-10 levels. However, an increase in the proinflammatory cytokine IL-1β and IL-6 levels was observed. As far as we are aware, few studies have been made on the effect of TNX in cell cultures. Nonetheless, piroxicam, a TNX analogue, has been encapsulated into liposomes showing, for instance, a reduction of TNF-α, IL-1β and PGE_2_ while increasing IL-10 in LPS-stimulated RAW264.7 [69].

CLX demonstrated to reduce not only COX-2 and PGE_2_ levels in different in vitro and in vivo models and in synovial fluid collected from OA patients, but also levels of several proinflammatory cytokines such as TNF-α, IL-1β and IL-6 [70,71]. Beside this anti-inflammatory action, recent in vitro and in vivo investigations suggest that CLX has additional disease-modifying effects such as chondroprotective effects, prevention of synovial hyperplasia or inhibition of bone destruction, that could slow OA disease progression [72]. The CLX-10 loaded NPs described here showed a reduction of all the proinflammatory factors TNF-α, IL-1β, IL-6 and PGE_2_, but also of IL-10.

Finally, DEX is a glucocorticoid whose anti-inflammatory potential is mainly mediated by its binding to the glucocorticoid receptor, which is activated and translocated to the nucleus of immune cells, inducing or repressing the transcription of multiple inflammation related genes [73]. Moreover, it can exert its anti-inflammatory effect by interfering with key inflammatory transcriptional regulators like NF-κB and AP-1, or by suppressing the enzyme phospholipase A2 (PLA2) and, therefore, the conversion of phospholipids into arachidonic acid [15]. Here, DEX-15 NPs achieved the highest inhibition of RAW264.7 release of all inflammatory factors with the exception of PGE_2_. As for CLX-10 NPs, IL-10 was also reduced, which may be due to the overall reduction of the inflammatory cascade.

Altogether, these loaded NPs are potential candidates for the treatment of OA since they were demonstrated to be in vitro modulators of the cellular release of different osteoarthritic inflammatory markers.

### 3.5. Histological Evaluation: In Vivo Biocompatibility

**Figure 6:**
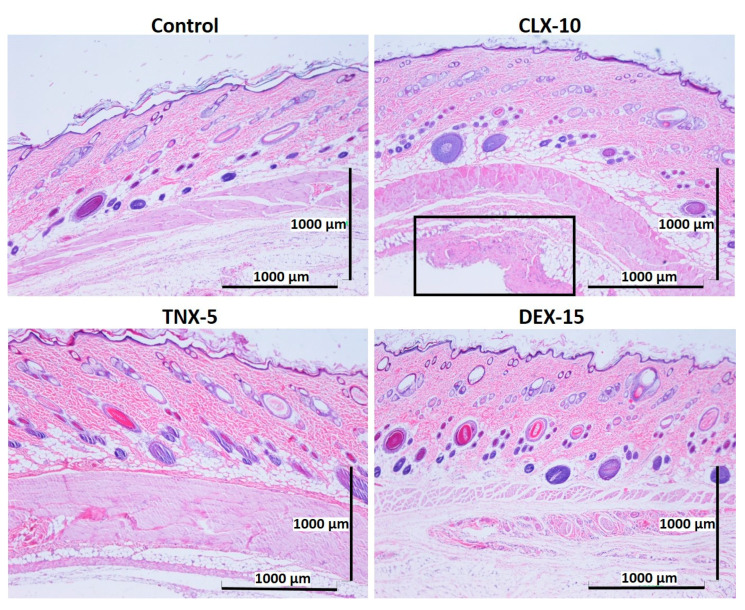
Representative histological photomicrographs of skin cross-sections of rats subcutaneously injected with CLX-10, TNX-5 and DEX-15 NPs compared to the control group after one week. In the image corresponding to CLX-10-treated rats, some tissue reaction (squared and amplified in Figure S5) was observed, mainly based on macrophage infiltration (H-E).

**Figure 7:**
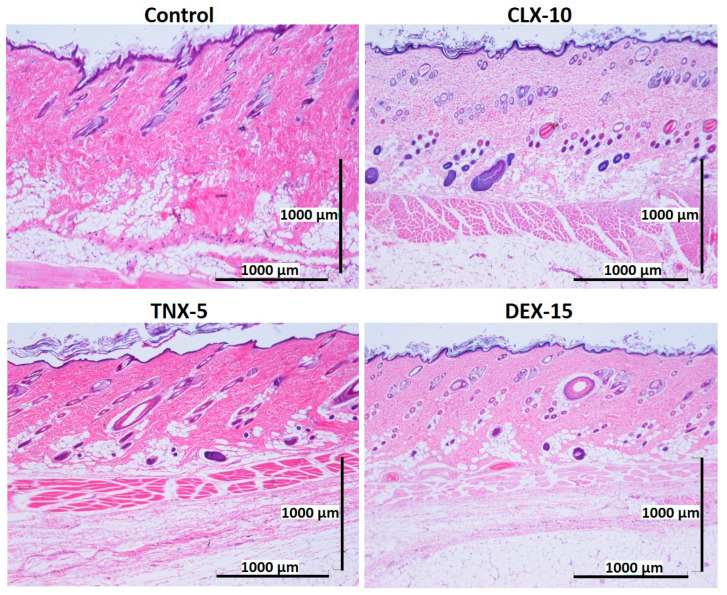
Representative histological photomicrographs of skin cross-sections of rats subcutaneously injected with CLX-10, TNX-5 and DEX-15-loaded NPs compared to the control group after two weeks. (H-E).

Biocompatibility is a prerequisite for the successful use of drug delivery systems in vivo. Here, NP in vivo biocompatibility was assessed by a subcutaneous injection of the loaded NPs (CLX-10, TNX-5 and DEX-15) in the dorsal of Wistar rats. The biocompatibility of unloaded NPs was demonstrated in a previous study [40]. Unloaded NPs evidenced no dermo-epidermal alterations and no inflammatory infiltrates. A control group containing PBS was evaluated for comparative purposes. The histological response of each group was evaluated after one and two weeks of the injection (Figure 6 and Figure 7).

After one (Figure 6) and two (Figure 7) weeks of implantation the control group did not show infiltration of inflammatory cells in the epidermis, dermis or deep muscle layers. Harvested tissues from these samples were evaluated as histologically normal. In the same way, rats treated with CLX-10, TNX-5 and DEX-15 NPs did not show tissue alterations. Visual observation of skin modification was not appreciated, and the epidermis, dermis and subcutaneous tissue appeared mostly intact. These tissues were histologically normal as in the control group after one and two weeks, except for CLX-10 NPs after the first week (squared at Figure 6). In this case, one of the rats treated with CLX-10 NPs showed a small connective tissue below the muscle layers (Figure S5). A reduced localized area of macrophage infiltration in the subcutaneous tissue was observed with small-congested vessels. This minimal inflammation was not maintained in time, since, after two weeks of the treatment inflammation was completely resolved. The recruitment of inflammatory cells, mainly macrophages, normally involves phagocytic processes. Scattered capillaries and smalls vessels were observed in one of the rats treated with CLX-10 NPs after one week. The presence of erythrocytes in the vessel lumina (Figure S5) suggested functional blood vessels. Moreover, the development of connective tissue, considered as a normal reaction, implies collagen synthesis and plays a crucial role in tissue repair [74]. Thereby, this occasional tissue reaction, which did not occur in the rest of the CLX-10 NPs-treated animals, could be attributed to the normal healing process of the injection.

The demonstrated in vitro and in vivo biocompatibility of the described NPs was confirmed firstly in vitro, both in HC-a and RAW264.7 cytotoxicity assays, and then in an in vivo subcutaneous injection model in rats, demonstrating the suitability of these systems for further in vivo models.

## 4. Conclusions

In this study, CLX, TNX and DEX-loaded NPs were successfully developed using an amphiphilic terpolymer nanovehicle. In all cases, NPs showed spherical morphologies, unimodal size distributions with sizes between 110 and 130 nm, and moderately negative charges between -1 and -5 mV. Encapsulation efficiencies were highly dependent of the drug water solubility, obtaining the highest values for CLX followed by TNX and DEX. In vitro cellular results demonstrated a wide range of noncytotoxic drug and NP concentrations and a reduction of the cytotoxicity of free CLX and DEX when nanoencapsulated. Loaded NPs showed an immunoregulatory effect on different osteoarthritic inflammatory markers in an LPS-stimulated RAW264.7 model by reducing the cellular release of NO, TNF-α, IL-1β, IL-6, PGE_2_ and IL-10 in the case of CLX and DEX NPs, having the strongest inhibition with DEX NPs for all the factors but PGE_2_. On the other hand, TNX NPs showed an inhibition of the release of NO and PGE_2_, although a significant stimulation of inflammatory markers IL-1β, IL-6 and IL-10 was evidenced. Lastly, the in vivo biocompatibility of the three loaded NPs in a NP subcutaneous injection rat model showed no histological differences between NP-treated and control rats after two weeks of injection. In conclusion, the suitable physicochemical characteristics, the in vitro anti-inflammatory activity and the in vivo biocompatibility properties of these NPs allow us to propose them as potential therapeutic agents of OA, as regulators of the cellular release of inflammatory factors. Taking the physicochemical and biological results overall, we consider that CLX NPs are the most optimal systems, in particular CLX-10 NPs at 0.50 mg mL^−1^, followed by DEX-15 NPs at 0.50 mg mL^−1^. It is worth saying that currently, further in vitro and in vivo animal models are being performed to fully elucidate the potential of these anti-inflammatory-loaded NPs as local intra-articular treatments of OA.

## Figures and Tables

**Table 1:** Summary of celecoxib (CLX), tenoxicam (TNX) and dexamethasone (DEX) properties related to the treatment of osteoarthritis (OA).

	Celecoxib	Tenoxicam	Dexamethasone
Type	Coxib, selective cyclooxygenase 2 (COX-2) Inhibitor	Oxicam, class of nonsteroidal anti-inflammatory drugs (NSAID)	Glucocorticoid, class of corticoid
Chemical structure	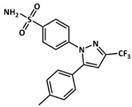	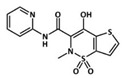	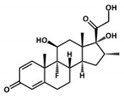
Main mechanism of action	Selective inhibition of COX-2	Inhibition of both COX-1 and COX-2	Binding to cellular glucocorticoid receptors, inducing or repressing the transcription of multiple genes
Solubility in water (mg L^−1^)	4.3 [41]	14.1 [42]	89.0 [43]
LogP (partition coefficient)	3.53 [41]	1.9 [42]	1.83 [43]
Most common administration route	Oral	Intra-articular injection, oral	Intra-articular injection, oral
Current used in OA	Inflammation and pain relief in mild or moderate OA	Post-operative pain relief	Inflammation and pain relief in mild or moderate OA

**Table 2:** Concentrations of feed and encapsulated drugs in NP suspensions (2.00 mg mL^−1^) in terms of % *w*/*w* with respect to the terpolymer and mg mL^−1^.

NP Code	[Drug]		EE(%)	[Encapsulated Drug]	
	% *w*/*w*	mg mL^−1^		% *w*/*w*	mg mL^−1^
CLX-5	5	0.10	72 ± 8	3.60 ± 0.29	0.072 ± 0.006
CLX-10	10	0.20	50 ± 7	5.00 ± 0.35	0.100 ± 0.007
CLX-20	20	0.40	39 ± 6	7.80 ± 0.47	0.156 ± 0.009
TNX-2	2	0.04	22 ± 5	0.44 ± 0.03	0.009 ± 0.001
TNX-5	5	0.10	20 ± 4	1.00 ± 0.07	0.020 ± 0.001
TNX-10	10	0.20	24 ± 5	2.50 ± 0.18	0.050 ± 0.004
DEX-10	10	0.20	26 ± 6	6.76 ± 0.34	0.052 ± 0.003
DEX-15	15	0.30	16 ± 7	0.80 ± 0.10	0.048 ± 0.002
DEX-20	20	0.40	14 ± 7	0.70 ± 0.10	0.056 ± 0.003

## Data Availability

Not applicable.

## Supplementary Materials

The following are available online at https://www.mdpi.com/1999-4923/13/2/290/s1, Figure S1: (A) Scheme of terpolymer poly(MVE-*co*-VP-*co*-VC) synthesis. (B) Terpolymer ^1^H-NMR spectrum (400 MHz) in CDCl_3_. (C) ^1^H-NMR, TGA, DSC and GPC characterization results of the terpolymer. Figure S2: NP stability in terms of aggregation; Figure S3: Representation of HC-a viabilities after 24 h of exposure to the NPs or to the corresponding free drug against the encapsulated concentrations of (A) CLX, (B) TNX and (C) DEX; Figure S4: Representation of RAW264.7 viabilities after 24 h of exposure to the NPs or to the corresponding free drug against the encapsulated concentrations of (A) CLX, (B) TNX and (C) DEX; Figure S5: Representative image of tissue section of rats treated with CLX-10 NPs 1 week post-injection; Table S1: Inflammatory factors studied in the mouse inflammation antibody array.

## Abbreviations

ADAMTS: A disintegrin and metalloproteinase with thrombospondin motifs; AIBN, azobisisobutyronitrile; ANOVA, analysis of variance; AP-1, activator protein 1; CNT, control; CLX, celecoxib; COX, cyclooxygenase; CUR, curcumin; DEX, dexamethasone; D_h_, hydrodynamic diameter; DLS, dynamic light scattering; DMEM, Dulbecco’s Modified Eagles Medium; DSC, differential scanning calorimetry; EE, encapsulation efficiency; ELISA, enzyme linked immunosorbent assay; FBS, fetal bovine serum; GPC, gel permeation chromatography; HC-a, human articular chondrocytes; H-E, hematoxylin and eosin; IL, interleukin; ^1^H RMN, proton nuclear magnetic resonance; LDE, Laser Doppler Electrophoresis; LPS, lipopolysaccharide; MCP, monocyte chemoattractant protein; MCSF, macrophage colony stimulating factor; MIP, macrophage inflammatory protein; MMP, matrix metalloproteinase; MVE, α-tocopheryl methacrylate; NF-κB, nuclear factor kappa B; NO, nitric oxide; NP, nanoparticle; NSAID, non-steroidal anti-inflammatory drug; OA, osteoarthritis; PBS, phosphate buffered saline; PDI, polydispersity index; PEG, poly ethylene glycol; PES, polyethersulfone; PG, prostaglandin; PLA2, phospholipase A2; PLGA, polylactide-*co*-glycolide; RANTES, Regulated upon Activation, Normal T cell Expressed, and Secreted; ROS, reactive oxygen species; SD, standard deviation; sTNF-R, soluble tumor necrosis factor receptor; TGA, thermogravimetric analysis; TIMP, tissue inhibitor of metalloproteinases 2; TLR, toll-like receptors; TNF-α, tumor necrosis factor-α; TNX, tenoxicam; UV, ultraviolet; VC, *N*-vinylcaprolactam; VP, 1-vinyl-2-pyrrolidone
