# Supplementary material for: Modulation of Inflammatory Mediators by Polymeric Nanoparticles Loaded with Anti-Inflammatory Drugs
Source: Pharmaceutics. 2021 Feb 23;13(2):290. doi: 10.3390/pharmaceutics13020290 (PMC7926915; doi:10.3390/pharmaceutics13020290)
Supplement: Supplementary file 1 [file pharmaceutics-13-00290-s001.pdf]

pharmaceutics

MDPI

Supplementary Material: Modulation of Inflammatory Mediators by Polymeric Nanoparticles Loaded with Anti-Inflammatory Drugs

Gloria María Pontes-Quero, Lorena Benito-Garzón, Juan Pérez Cano, María Rosa Aguilar and Blanca Vázquez-Lasa

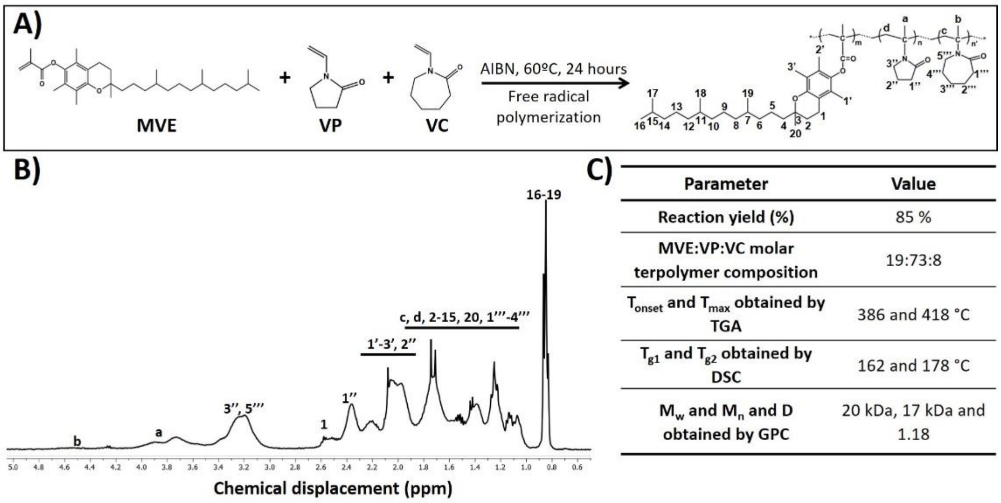

Parameter	Value
Reaction yield (%)	85 %
MVE:VP:VC molar terpolymer composition	19:73:8
T_{onset} and T_{max} obtained by TGA	386 and 418 °C
T_{g1} and T_{g2} obtained by DSC	162 and 178 °C
M_w and M_n and D obtained by GPC	20 kDa, 17 kDa and 1.18

Figure S1. (**A**) Scheme of terpolymer poly(MVE-*co*-VP-*co*-VC) synthesis. (**B**) Terpolymer ^{1}H-NMR spectrum (400 MHz) in $CDCl_3$. (**C**) ^{1}H-NMR, TGA, DSC and GPC characterization of the terpolymer. T_{onset}, temperature at the onset of the major decay; T_{max}, maximum thermal degradation rate temperature; T_g, glass transition temperature; M_w, weight average molecular weight; M_n, number average molecular weight, Đ, polydispersity index.

Citation: Pontes-Quero, G.M.; Benito-Garzón, L.; Pérez Cano, J.; Aguilar, M.R.; Vázquez-Lasa, B. Modulation of Inflammatory Mediators by Polymeric Nanoparticles Loaded with Anti-Inflammatory Drugs. **2021**, *13*

Publisher's Note: MDPI stays neutral with regard to jurisdictional claims in published maps and institutional affiliations.

Copyright: © 2021 by the authors. Submitted for possible open access publication under the terms and conditions of the Creative Commons Attribution (CC BY) license (http://creativecommons.org/licenses/by/4.0/).

A)

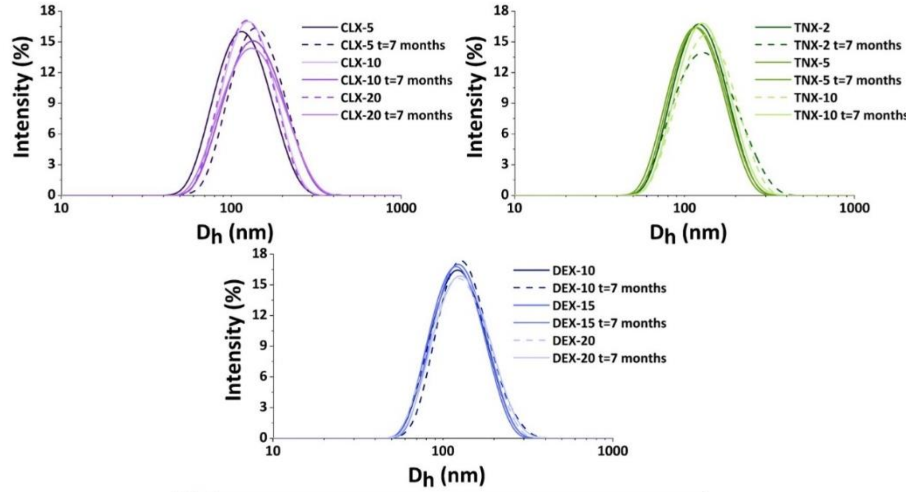

B)

NP code	D_h (nm)	PDI
**CLX-5**	120.3 ± 2.4	0.162 ± 0.011
**CLX-10**	130.2 ± 1.5	0.109 ± 0061
**CLX-20**	126.1 ± 1.5	0.109 ± 0.061
**TNX-2**	124.4 ± 0.6	0.139 ± 0.011
**TNX-5**	116.8 ± 1.2	0.139 ± 0.019
**TNX-10**	122.4 ± 1.7	0.085 ± 0.027
**DEX-10**	136.3 ± 2.2	0.182 ± 0.014
**DEX-15**	123.3 ± 1.2	0.143 ± 0.017
**DEX-20**	125.8 ± 2.0	0.139 ± 0.024

Figure S2. NP stability in terms of aggregation. (**A**) NP size distribution assessment by dynamic light scattering immediately after synthesis and after 7 months of static storage at 4 °C. (**B**) Hydrodynamic diameter (D_h) and polydispersity index (PDI) of loaded NPs after 7 months of static storage at 4 °C.

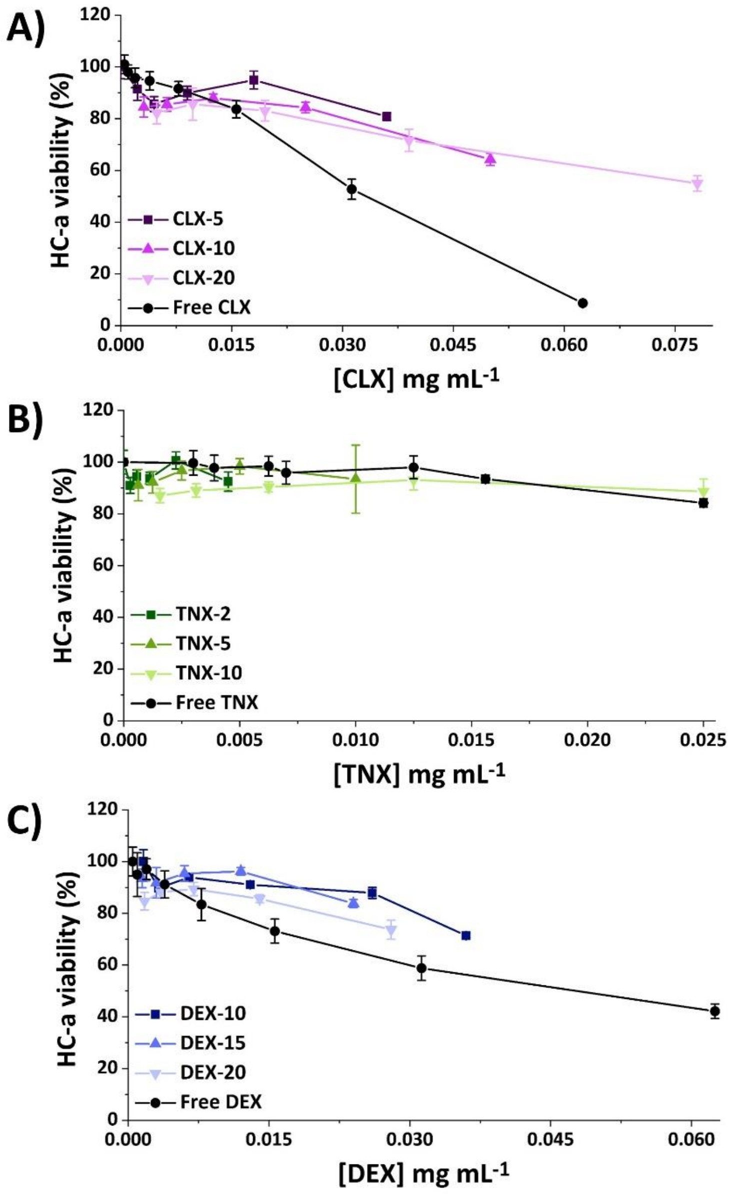

Figure S3. Representation of HC-a viabilities after 24 h of exposure to the NPs or to the corresponding free drug against the encapsulated concentrations of (**A**) CLX, (**B**) TNX and (**C**) DEX.

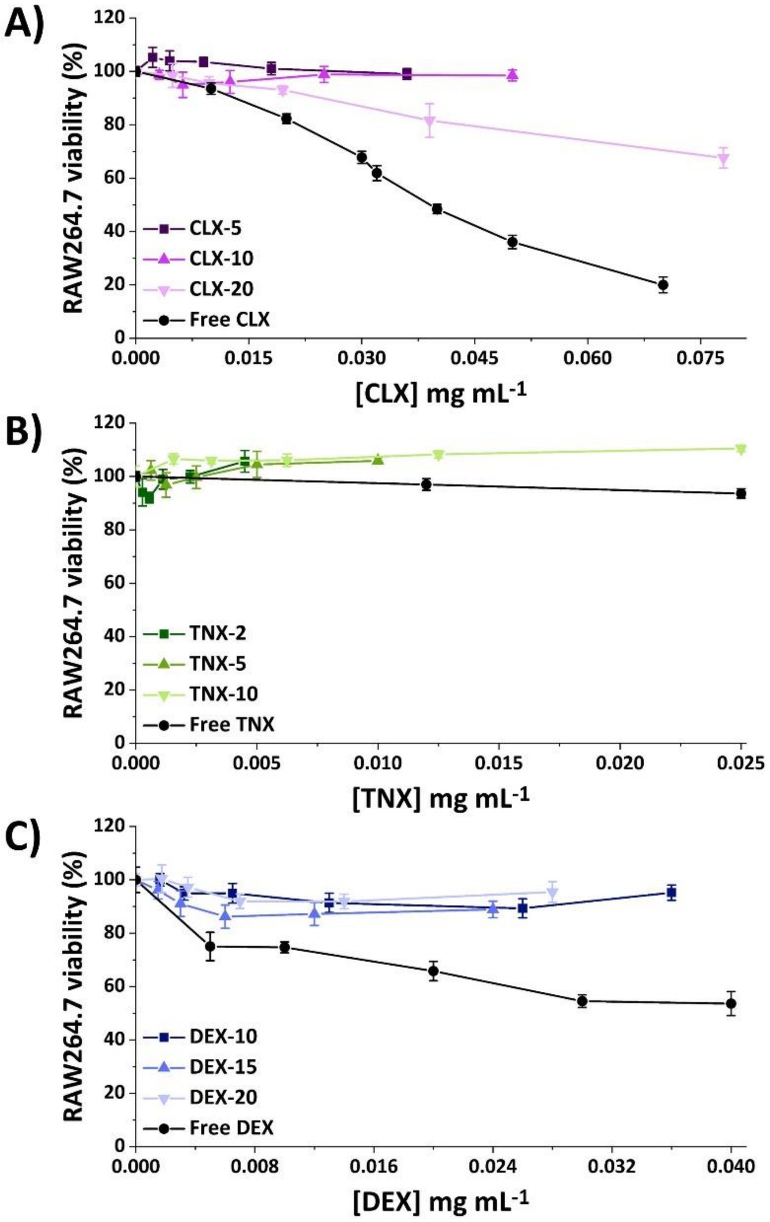

Figure S4. Representation of RAW264.7 viabilities after 24 h of exposure to the NPs or to the corresponding free drug against the encapsulated concentrations of A) CLX, B) TNX and C) DEX.

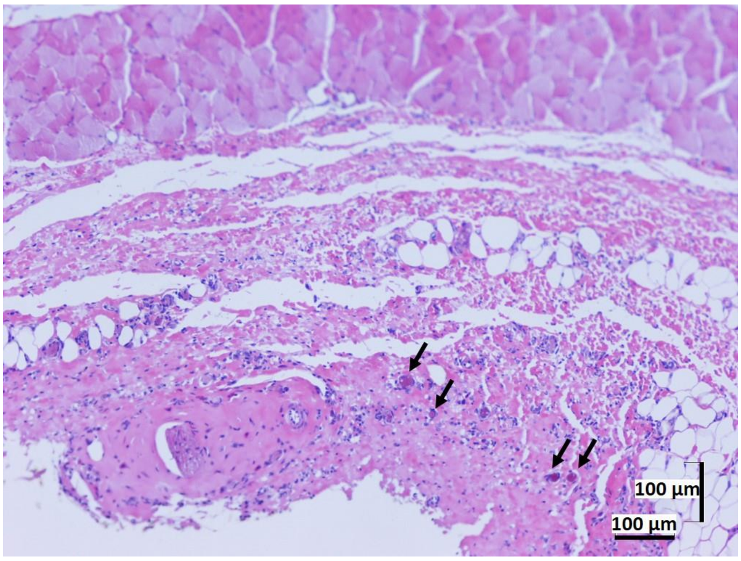

Figure S5. Representative image of tissue section of rats treated with CLX-10 NPs 1 week after injection. A small inflammatory reaction was appreciated mainly based on macrophage infiltration at the subcutaneous tissue. Small vessels are highlighted with arrows. (H-E, scale bar: 100 µm).

Table S1. Inflammatory factors studied in the mouse inflammation antibody array.

**Inflammatory factors under study**
BLC, CD30L, Eotaxin, Eotaxin-2, Fas Ligand, Fractalkine, GCSF, GM-CSF, IFN-ϒ, IL-1α, IL-1β, IL-2, IL-3, IL-4, IL-6, IL-9, IL-10, IL-12 p40/p70, IL-12 p70, IL-13, IL-17, I-TAC, KC/CXCL1, Leptin/OB, LIX, Lymphotactin, MCP-1, MCSF, MIG, MIP-1α, MIP-1ϒ, RANTES, SDF-1, TCA-3, TECK, TIMP-1, TIMP-2, TNF-α, sTNF RI, sTNF-RII
